# Coherent apoptotic and autophagic activities involved in regression of chicken postovulatory follicles

**DOI:** 10.18632/aging.101436

**Published:** 2018-04-29

**Authors:** Xin Lin, Xingting Liu, Yanfen Ma, Yuling Mi, Weidong Zeng, Jian Li, Caiqiao Zhang

**Affiliations:** 1 Department of Veterinary Medicine, College of Animal Sciences, Zhejiang University, Hangzhou, Zhejiang Province 310058, China

**Keywords:** postovulatory follicle, regression, mitochondrial apoptosis, endoplasmic reticulum stress, autophagy

## Abstract

After ovulation in mammals, rupture of mature follicles is reorganized into the corpus luteum that secrets progesterone (P_4_) to stimulate endometrial development. The situation in birds differs considerably. Beyond ovulation the ruptured avian follicle forms a postovulatory follicle (POF) that is not considered analogous to mammalian corpus luteum. The function and regression mechanisms of avian POFs remain poorly understood. Here we investigated the changes in apoptotic and autophagic activities that were involved during POF degradation. Results showed that the structure and secretory function of POF3 manifested the most apparent deterioration during whole processes of regression. A TUENL assay revealed that the granulosa layer maintained longer viability than the theca layer. Importantly, mitochondrial apoptosis and endoplasmic reticulum (ER) stress-associated genes and proteins reached their highest levels in the granulosa cells of POF3. Beclin1 was distributed mainly in theca cells and coupled with LC3β-II accumulation, Sequestosome-1 (p62) degradation and Beclin1 elevation confirmed that autophagic activity had increased dramatically in the theca layer of POFs. These results indicate that the apoptosis of the granulosa cells from POFs occurs by mitochondrial apoptosis and ER stress and that a coherence of Beclin1-induced autophagy and caspase-induced apoptosis results in regression of theca layers of avian POFs.

## Introduction

In the chicken, the postovulatory follicle (POF) forms from the largest preovulatory follicle (F1). Although the POF, containing both granulosa and theca layers, plays an important role in oviposition and nesting behavior [1], there is a consensus that it has no structure or function directly analogous to that of the corpus luteum in mammals [2]. The POFs suffer structural and functional regression after ovulation and this process is almost entirely completed within a 4-6 day period [3–5]. Caspase-induced apoptosis is known to impose a pivotal impact on the regression of POFs [4,6]. However, beyond apoptosis, few studies have reported any further details regarding any other aspects of the molecular mechanisms involved in the regression of the avian POFs.

Multiple endogenous and exogenous upstream signals regulate apoptosis prior to the activation of caspase3 and subsequent internucleosomal DNA fragmentation [7]. One of the most important pathways of endogenous apoptotic signals is known to activate the mitochondrial pathway. The B-cell lymphoma-2 (BCL2) family of proteins, including anti-apoptotic (BCL2, B cell lymphoma/leukemia X (BCLX), etc.) and pro-apoptotic proteins (BCL2-associated X protein (BAX), BCL2-related ovarian killer (BOK), BCL2 homologous antagonist killer (BAK), etc.), are key regulators that are located in the mitochondrial membrane. Their interactions control the release of cytochrome c [8]. The positively correlation between mitochondria-mediated apoptosis and the regression of the spontaneous atresia follicles has been well documented and granulosa cell apoptosis is known to depend on the expression of BCL2 or Bax [9]. However, whether these proteins contribute to the regression of POFs still remains uncertain.

The endoplasmic reticulum (ER) is another essential organelle responsible for both the synthesis and the folding of proteins, the trafficking and metabolism of lipids and sterols and the control of cellular Ca^2+^ storage [10]. Accumulating evidence suggests that cellular apoptosis is initiated by excessive or persistent ER stress. The role of glucose-regulated protein 78 (GRP78) seems to be central to the regulation of the unfolded protein response and is used as a primary marker for detecting the induction of ER stress. GRP78 activates three downstream ER stress signaling pathways: (i) inositol-requiring enzyme 1α (IRE1α) - X-box binding protein-1 (XBP1), (ii) protein kinase RNA (PKR)-like ER kinase (PERK) - activating transcription factor-4 (ATF4)-C/EBP-homologous protein (CHOP), (iii) activating transcription factor 6α (ATF6α) [11]. Furthermore, BCL2 is involved in ER stress and participates in anti-apoptosis mechanisms. This highlights that ER stress may play an indirect role through interaction with mitochondria-mediated molecular signaling pathways [12]. In addition, ER stress is a risk factor associated with steatosis resulting from nonalcoholic steatohepatitis [13]. Related studies indicate that ER stress may be involved in the regression of the POFs.

Apoptosis is not the only mechanism involved in the granulosa and theca cell death during POF cellular regression. In fact, in mammals the majority of the ovarian cells are eliminated by non-apoptotic mechanisms such as autophagy and necrosis, during natural or induced follicular regression [14,15]. The process of autophagy begins with formation of double-membrane vesicular structures that encircle various organelles and parts of the cytoplasm [16]. Beclin1 and microtubule-associated proteins 1A/1B light chain 3B (LC3β) are key proteins regulating both autophagy and cell death. Moreover, sequestosome-1 (SQSTM1/p62) is a protein targeting the specific cargo of autophagy [17]. Beclin1 can also interact with the anti-apoptotic protein BCL2, where its binding state plays a key part in coordinating the cellular decision to undergo autophagy [18]. At the molecular level, the crosstalk between apoptosis and autophagy is manifested by numerous genes that are shared by both pathways. However, the impact of autophagy, as involved in regression of the POFs, has received little attention.

Since apparent differences exist between the regression of the mammalian corpus luteum and avian POFs, the objective of this study was to investigate the mechanism of POF regression in the chicken. The coherent changes in the apoptotic and autophagic activities were evaluated from POFs at different stages of regression to elucidate the molecular and cellular cues leading to progressive degradation of the POFs in the chicken.

## RESULTS

### Morphology of the regressed POFs

**Figure 1:**
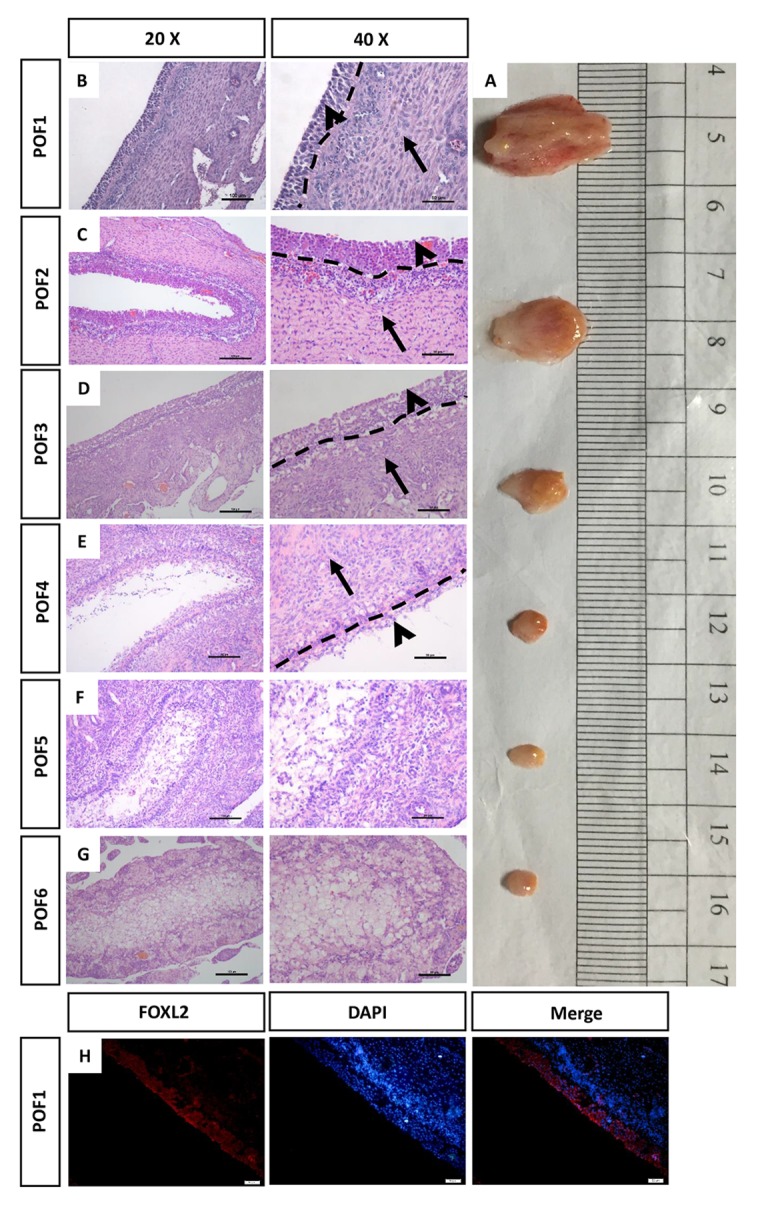
**Morphology of the regressed POFs.** (**A**) The removal of POFs (POF1-POF6). (**B**-**G**) HE staining was used to evaluate the morphology of POFs. The granulosa layer (arrowheads) and the theca layer (arrows) from POF1 to POF4 were separated by a dashed line. (These were indistinguishable in POF5 and POF6). Scale bars: 100 μm (20×) and 50 μm (40×). (H) Histological sections of POF1 were given an immunofluorescent label with granulosa cell marker FOXL2 (Red), where granulosa cells were mainly distributed in the granulosa layer. Scale bar: 50 μm.

After the chickens had laid six eggs in a sequence, POF1 (the most recently ruptured follicle) to POF6, identified by their different diameters, were isolated from their functional left ovaries (Fig. 1A). Hematoxylin & eosin (HE) stains were used to reveal the morphological structure of the POFs. The relative integrity of the granulosa layer was observed from POF1 to POF3 (Fig. 1B-D). By POF4, the majority of granulosa cells had degraded (Fig. 1E). Ruptured follicles were gradually reorganized after POF3, a process involving the coalescence of numerous physaliphore-like cells (Fig. 1F). The width of external thecal layers had reduced distinctly from POF1 to POF3 (Fig. 1A-D). There was no significant difference in the theca layer from POF4 to POF6 (Fig. 1A, E-G). FOXL2 box L2 (FOXL2, one of granulosa cell markers) was used to distinguish the granulosa layer from the POFs (Fig. 1H).

### Steatosis-like morphology in the granulosa cells from POFs

**Figure 2:**
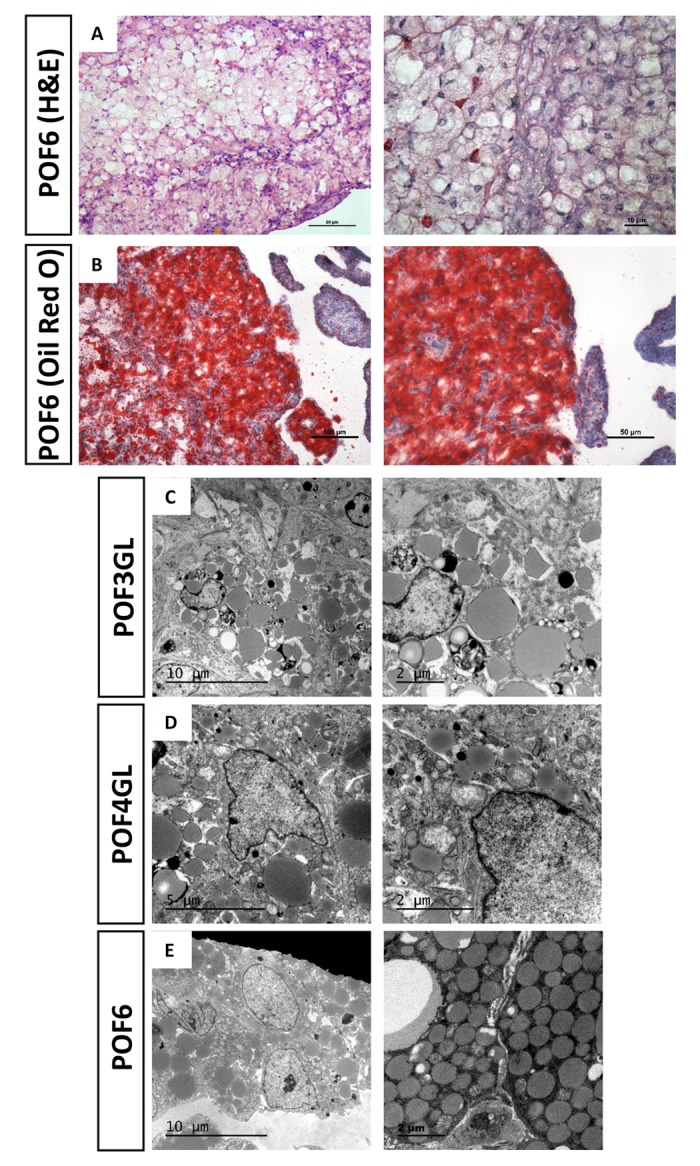
**Steatosis-like morphology occurring in the granulosa cells from POFs.** (**A**) HE staining was used to observe steatosis-like granulosa cells in POF6. Scale bars: 50 µm and 10 μm. (**B**) Oil Red O staining was used to verify lipid droplets existing in the coalescence of POF6. Scale bars: 100 µm and 50 µm. (**C**-**E**) TEM was used to observe the large quantity of lipid droplets existing in POF3, POF4 and POF6.

Figure 2A reveals numerous physaliphore-like cells in which vesicles are large enough to distort the nucleus in POF6 (Fig. 2A). We used Oil Red O staining and transmission electron microscopy (TEM) to detect the coalescence from the POF6. The results showed that an increasing number of lipid droplets occurred in the cytoplasm, thereby verifying that typical characteristic of steatosis-like morphology occurred in the coalescence of the POF6 (Fig. 2B, E). Moreover, we also detected the granulosa cells from the POFs (POF1-POF4) and observed that whilst few lipid droplets existed in POF1 and POF2, there was a striking changes were a large quantity of lipid droplets appeared in the cytoplasm of granulosa cells from POF3 onwards (Fig. 2C-D). The abnormal accumulation lipid droplets in the cytoplasm of granulosa cells seemed to be associated with POF degradation.

### Secretion of progesterone from POFs

**Figure 3:**
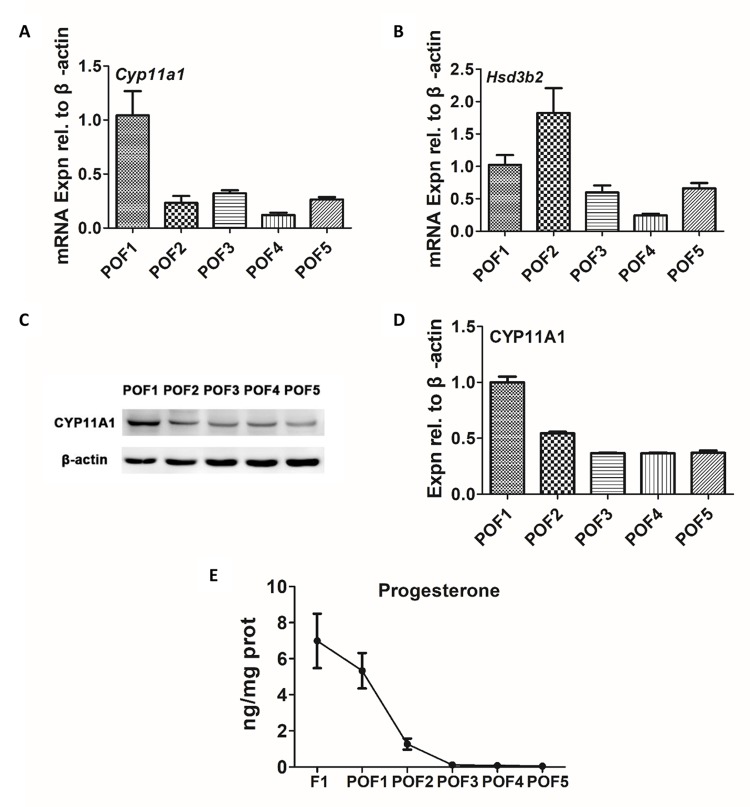
**Secretion of progesterone from POFs.** (**A**-**B**) qRT-PCR analysis of *Cyp11a1* and *Hsd3b2* mRNA abundance in POFs (POF1 to POF5). (**C**-**D**) WB analysis of CYP11A1 expression in the POFs (POF1 to POF5). (**E**) The level of P_4_ showed in F1 and POFs (POF1 to POF5). Values are means ± SEM of three experiments.

It has been previously reported that the granulosa cells of the POFs, especially the most recently ruptured follicle, are involved in steroidogenesis [19]. We firstly aimed to detect the expression of steroidogenic enzymes. While some POFs (POF1 to POF5) were able to express *Cyp11a1* and *Hsd3b2* at mRNA levels (Fig. 3A-B), the trends were unclear and we were unable to draw any definite conclusions. However, the protein level of CYP11A1 was initially strong but exhibited a rapid decline from POF1 to POF3, followed by a low but steady stage after POF3 (Fig. 3C-D). A similar result was observed for progesterone (P_4_) levels. The secretory capacity of P_4_ diminished greatly till POF3 (Fig. 3E).

### Caspase-induced apoptosis leads to POF degradation

**Figure 4:**
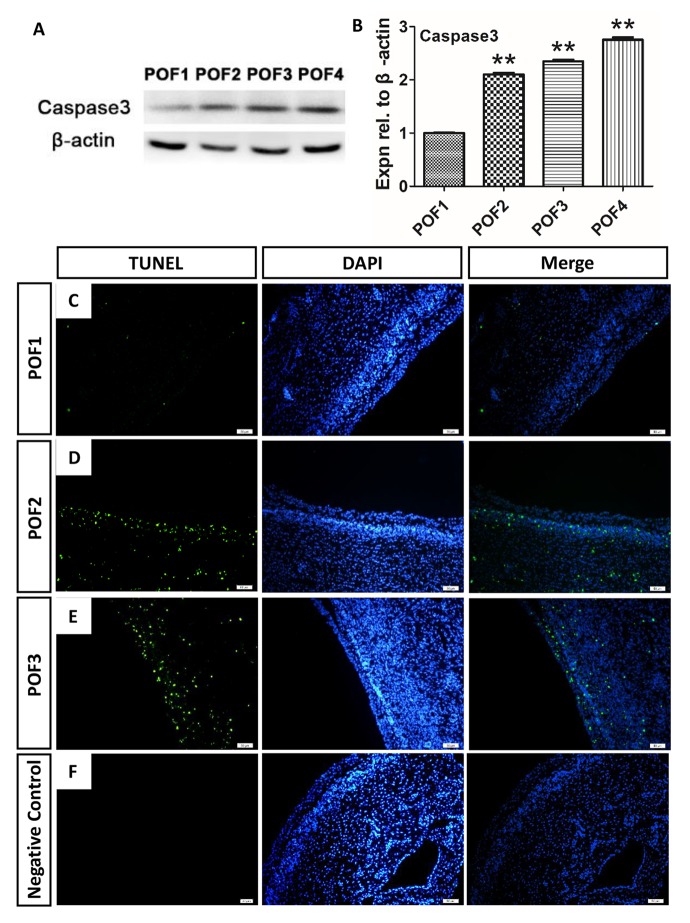
**Caspase-induced apoptosis leads to POF degradation.** (**A**-**B**) WB and grey analysis of caspase3 expression in POFs (POF1 to POF4). Values are means ± SEM of three experiments. Asterisks indicate significant differences (* *P*<0.05 and ** *P*<0.01). (**B**-**E**) Few TUNEL (green) marker labeled occurred in cells from POF1 to POF3. (**D**) Negative Control. Scale bar: 50 µm.

Consistent with the previous report in chickens that suggested that POF regression occurred via the process of programmed cell death [6], we found the caspase3 level was upregulated significantly from the basal level during POF regression (Fig. 4A-B). Furthermore, we used the TUNEL method to observe where the cell apoptosis was distributed. Interestingly, the degradation rate of the granulosa layer differed from that of the theca layer. Few TUNEL-labeling cells were present in either the granulosa or theca layers in POF1 (Fig. 4C). However, while there were few TUNEL-positive cells in the granulosa layer of POF2, numerous positive cells existed in the theca layer (Fig. 4D). Moreover, large number of positive cells occurred in both the granulosa and the theca layer of POF3 (Fig. 4E).

### Mitochondrial anti-apoptosis and ER stress was mainly distributed in the granulosa layer of POFs

**Figure 5:**
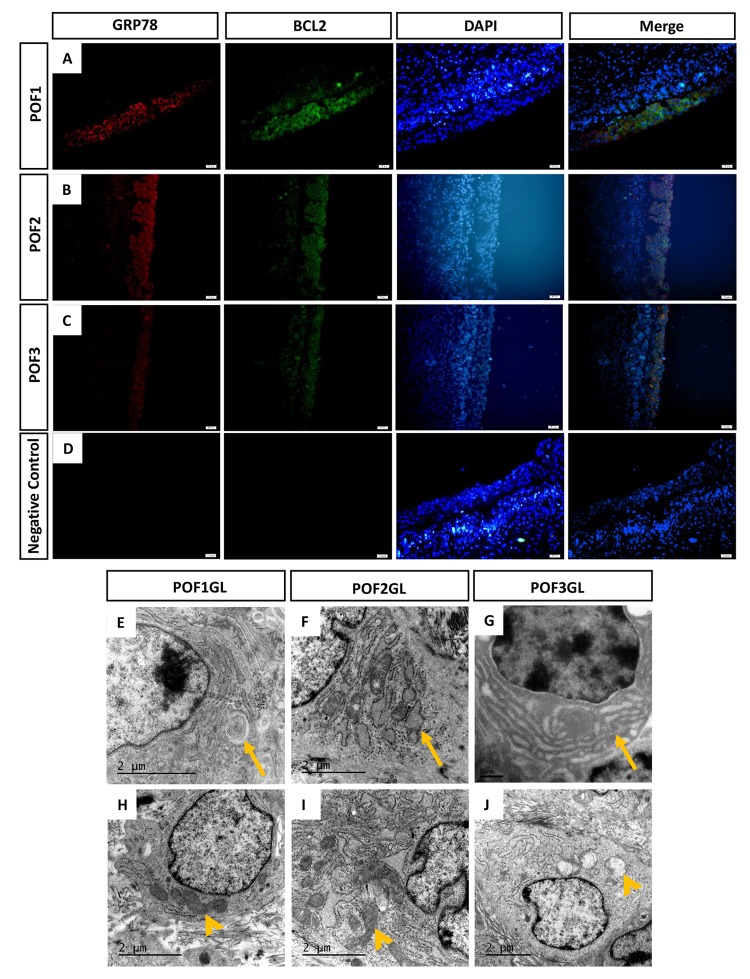
**GRP78 and BCL2 co-expressed in the granulosa layer from POFs.** (**A**-**C**) Histological sections of POFs (POF1 to POF3) were given immunofluorescent labels with ER stress marker GRP78 (Red) and mitochondria anti-apoptosis marker BCL2 (Green), showing the main distribution in the granulosa layer. Scale bar: 20 μm. (**D**) Negative Control. (**E**-**F**) TEM was used to observe ER lesions, concentric round, dilatation and vesiculation and hyperplasia, respectively (arrows). (**H**-**J**) TEM was used to observe normal mitochondria and vacuolated mitochondria, respectively (arrowheads). GL represents the granulosa layer.

As we found the number of lipid droplets accumulating after POF3 (Fig.2B), we speculated that this lipid droplet accumulation may be involved in the blocking of lipid synthesis-induced ER stress. In addition, few cells from POF1 or from the granulosa layer of POF2 were labeled by TUNEL. This suggests that some cellular anti-apoptotic mechanisms exist in POF1 and in the granulosa layer of POF2. The results from immunofluorescence (IF) staining showed that GRP78, one of marker proteins of ER stress, and BCL2, a marker for mitochondrial apoptosis inhibition, were co-expressed in the granulosa layer of POF1-3 (Fig. 5A-D). TEM observation revealed various degrees of ER lesions such as concentric round (Fig. 5E), dilatation and vesiculation (Fig. 5F) and hyperplasia (Fig. 5G) in the granulosa cells from POF1 to POF3. While very few abnormal mitochondria appeared in the granulosa cells from POF1 and POF2 (Fig. 5H-I), vacuolization in many mitochondria was observed in the granulosa cells from POF3 (Fig. 5J).

### Granulosa cells of POF3 suffer apparent mitochondrial apoptosis and ER stress

**Figure 6:**
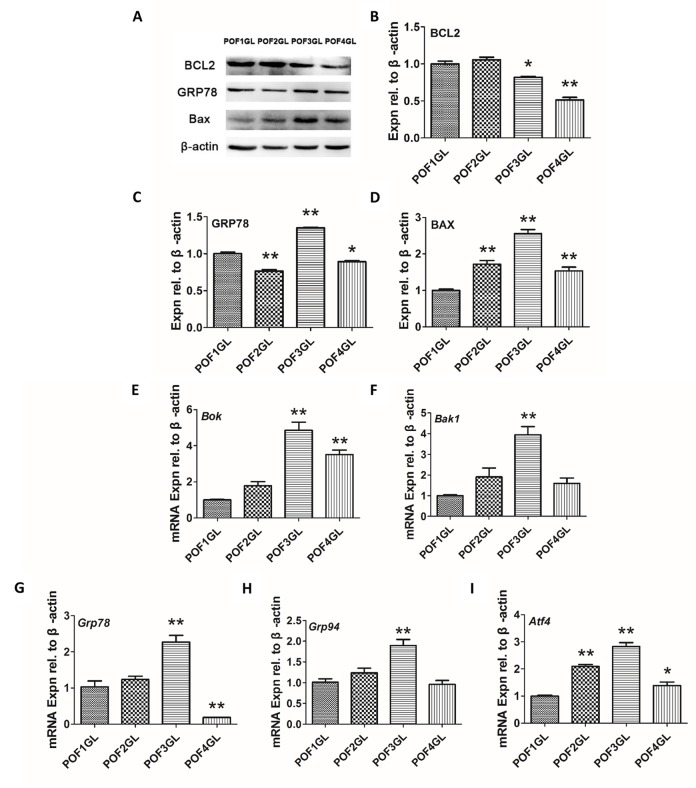
**Granulosa cells from POF3 suffer apparent mitochondrial apoptosis and ER stress.** (**A**-**D**) WB and grey analysis of BCL2, GRP78 and BAX expression in the granulosa layer from POFs (POF1 to POF4). (**E**-**I**) qRT-PCR analysis of *Bok*, *Bak1, Grp78*, *Grp94* and *Atf4* mRNA abundance in the granulosa layer from POFs (POF1 to POF4). GL represents the granulosa layer. Values are means ± SEM of three experiments. Asterisks indicate significant differences (* *P*<0.05 and ** *P*<0.01).

As IF results revealed that GRP78 and BCL2 were co-expressed in the granulosa layer of POFs, abnormal mitochondria appeared in POF3 and that ER lesions occurred from POF1 to POF3, we further conducted quantitative analysis by Western blot (WB) and qRT-PCR. Although the granulosa layer from POF1 to POF3 expressed BCL2 (Fig. 5A-D), the protein level of BCL2 had apparently decreased from POF1 to POF4 (Fig. 6A-B). Furthermore, the granulosa cells maintained higher expressions of GRP78 and BAX (one of mitochondrial pro-apoptotic proteins) in POF3 than that in any other POFs stages (Fig. 6A, C-D). In line with the results of the WB, mRNA expression revealed a similar trend where two mitochondrial pro-apoptotic related genes (*Bok* and *Bak1*, with high sequence similarities to *Bax*) and three ER stress related genes (*Grp78*, *Grp94* and *Atf4*) reached a peak expression in the granulosa layer of POF3 (Fig. 6E-I).

### Autophagy occurs in the theca layer of POFs

**Figure 7:**
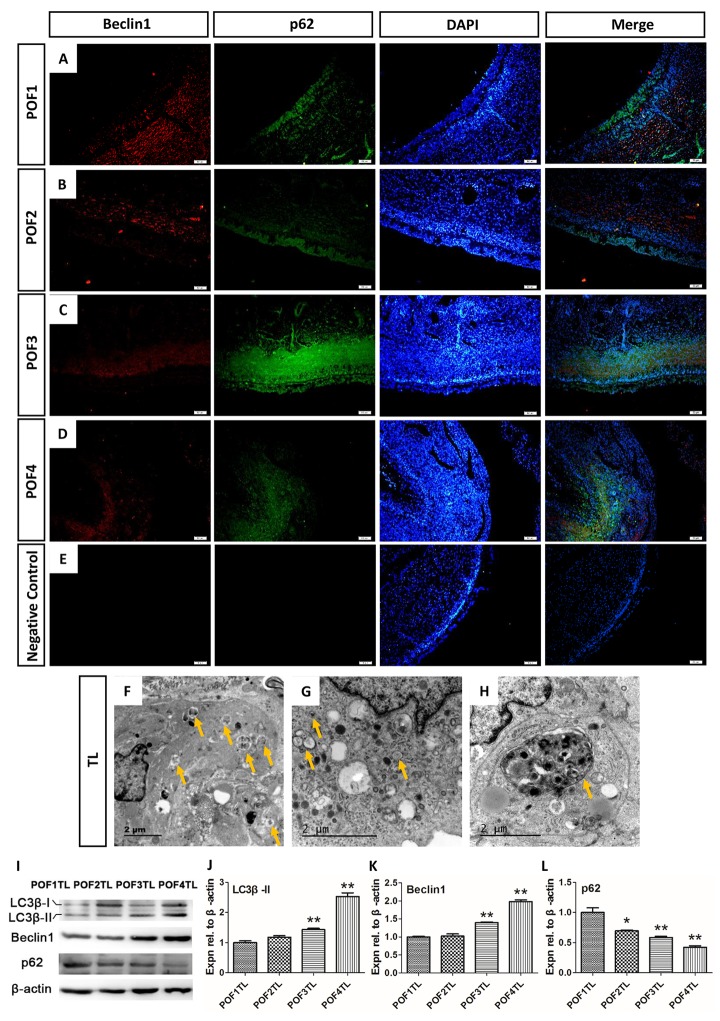
**Autophagy mainly occurred in the theca layer of the POFs.** (**A**-**D**) Histological sections of POFs (POF1-POF4) were given an immunofluorescent label with the autophagy marker Beclin1 (Red), which was mainly distributed in the theca layer, and p62 (Green). Scale bar: 50 μm. (**E**) Negative Control. (**F**-**H**). TEM was used to observe the large amounts of autophagosomes and autolysosomes existing in the theca layer of the POFs (arrows). (**I**-**L**) WB and grey analysis of LC3β-II, Beclin1 and p62 expression in POFs (POF1 to POF4). TL represents the theca layer. Values are means ± SEM of three experiments. Asterisks indicate significant differences (* *P*<0.05 and ** *P*<0.01).

Beclin1 exerts a great influence on the formation of autophagy and p62 serves as an ubiquitin-binding autophagy receptor. Images from IF showed that Beclin1 was mainly distributed in the theca layer among POFs (Fig. 7A-E) and that p62 was expressed in the granulosa layers in POF1 as well as POF2 (Fig. 7A-B), but expressed in both the granulosa and theca layers of POF3 and POF4 (Fig. 7C-D). Moreover, TEM results confirmed that numerous autophagosomes and autolysosomes existed in the theca layer (Fig. 7F-H). In addition, results from WB verified that the level of two autophagy associated proteins (LC3β-II and Beclin1) had risen significantly, and p62 declined, with the POF’s regression (Fig. 7I-L).

## DISCUSSION

The mechanisms of chicken POF regression remain poorly understood. Traditionally, structural regression of the POFs has been simply considered a result of programmed cell death [6]. Using HE staining, we observed the loss of the majority of granulosa cells by POF4, and, consistent with the previous study, that the size of theca layer shrank dramatically after POF3 [20]. However, in our results the accumulation of discrete lipid droplets in granulosa cells appeared at a later stage than was reported in the previous study [20]. The TEM observation and Oil Red O staining verified that the accumulation of lipid droplets in the cytoplasm and such accumulation first appeared in POF3. This suggested that steatosis-like morphology actually began at POF3, despite the more obvious and typical steatosis-like morphology occurring in the coalescence of POF6. Consistent with the previous report, the caspase3 level did ascend significantly with POFs regression [7]. On the other hand, functional regression of POFs embodies a gradual loss of secretion capacity. The secretory function of POFs, especially the most recent ruptured follicle, must not be overlooked in the chicken ovary, as such secretions exert effects on oviposition and nesting behavior [1]. Previous studies showed that the level of P_4_ in the granulosa and theca layer descended significantly within 52 h [21]. Our studies revealed that the level of CYP11A1 decreased sharply at POF3 stage and that the biosynthesis of P_4_ is rapidly terminated in POF3. These results suggest that POF3 suffers that maximal apoptosis degree, at structural and functional levels, than any other POF level.

By using HE staining and TEM methods carried out on the chicken POFs, we have demonstrated steatosis-like morphology in the coalescence of POF6 [20]. Similar morphology has previously been found in hepatic steatosis due to fatty liver or nonalcoholic fatty liver disease [22]. Multiple factors can lead to hepatic steatosis and it is difficult to distinguish between the various causes. Intriguingly, Wyburn et al. observed that 24 h after ovulation, the granulosa cells were filled with agranular reticulum [23]. What’s more, increased lipid content in the steatotic liver induces chronic ER stress, mainly via the alteration of Ca^2+^ homeostasis [13]. As a result, superabundant lipid synthesis in granulosa cells is likely to be connected with smooth ER stress. Our results demonstrated that the number of smooth ERs did not increase significantly in the ultrastructure, despite ER stress being confirmed to occur after ovulation. We only found the pathological changes in their different extents occurring in the rough ER. The specific reason why steatosis-like morphology occurred in the granulosa cells from POFs needs further exploration.

As we have described above, it was unexpected that the granulosa layer maintained its biological functions longer than the theca layer. Gilbert et al revealed that the integrity of the granulosa layer was crucial for involvement of POF1 in oviposition [24]. It need further confirmation whether the granulosa cells from POF2 or older POFs still remain supplementary roles in secretory capacities before their complete morphological regression. In addition, both GRP78 and BCL2 were co-expressed within three days after ovulation, but the level of BCL2 decreased significantly from POF1 to POF4. This suggests that the anti-apoptosis ability of the mitochondria may be lost gradually rather than abruptly. Moreover, TEM observation demonstrated that the number of abnormal mitochondria increased in granulosa cells from POF3 onwards, which corresponded to the expression of the BAX protein and *Bok* and *Bak1* mRNA rising to their maximal levels. Similar results were revealed by examining ER stress markers. The expression of the GRP78 protein and *GRP78*, *Grp94* and *Atf4* mRNA also came up to their peak levels in POF3. This seems to strengthen the hypothesis that the granulosa cells of POF3 suffer severest apoptosis within the whole processes of regression.

In mammals, after ovulation the ruptured follicle is reorganized into the vascularization of corpus luteum. This process is directed by angiogenic factors such as vascular endothelial growth factor (VEGF) [25] and basic fibroblast factor (bFGF) [26] and is triggered by luteinizing hormone (LH). The corpus luteum, consisting of the granulosa-lutein cells and the theca-lutein cells, is involved in the production of relatively high levels P_4_ and relaxin which are required to prepare the uterus for embryo implantation and the maintenance of pregnancy. Whilst the rupture of chicken preovulatory follicles does not lead to the formation of anything resembling the corpus luteum [2], it has been found that POFs, especially the most recently ruptured follicle, are also able to secrete steroid hormones [21], prostaglandins [27] and relaxin [28]. Despite the differences in structure and function, the mechanisms of mammalian corpus luteum regression can therefore be compared to that of POFs. In mammals, the induction of autophagy and apoptosis in the regressing corpus luteum has been well described [14,29], with accumulating evidence that non-apoptotic forms of programmed cell death, such as autophagy, may also be involved [15,30]. Our results found not only that Beclin1 was mainly expressed in the theca externa of POFs, but also that numerous autophagosomes and autolysosome were directly observable in the theca cells. This implies that autophagy also provides important impacts on regression of the theca layer from POFs. WB analysis of the levels of LC3β-II accumulation, p62 degradation and Beclin1 elevation confirmed that the level autophagy increased dramatically with the regression of the theca layer from POFs, thereby indicating that the regressed theca layer relies largely on cell autophagy in addition to apoptosis.

At the cellular level, BCL2 is likely to play a pivotal role in controlling the balance between adaption and regression. Three aspects are of particular note. In the first aspect, BCL2 is able to regulate the function of the ER. Specifically, JNK-induced apoptosis involves the pro-apoptotic BAX and BAK which, in turn, are able to amplify the IRE1α signal [31]. Mechanisms of CHOP-induced apoptosis involve the suppression of the pro-survival protein BCL2 [32]. BOK has a proapoptotic function in regulating ER stress-induced apoptosis. This links apoptotic sensing at the ER to apoptotic activity at the mitochondria [33,34]. In the second aspect, BCL2 has a crucial effect on autophagy. BCL2 interacts with Beclin1 and downregulates Beclin1-dependent autophagy via PI3K-AKT-mTOR signaling [35]. Meanwhile, BCL2, cooperates with Atg5, a precursor for the synthesis of autophagosomes, to regulate both apoptosis and autophagy. In the third aspect, when ER stress occurs, GRP78 is released, after which PERK, IRE1α, and ATF6 activated their respective transducers [36]. XBP-1 also can trigger autophagy through the transcriptional activation of Beclin1 [37]. These aspects indicate that BCL2 may exert influence on the regression of POF.

**Figure 8:**
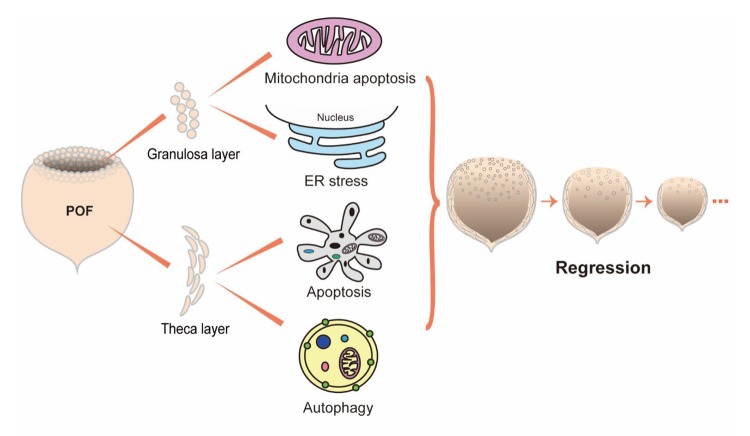
**Proposed model for the regression of POFs.**

In summary, this study demonstrates that chicken POFs regressed most apparently by POF3 after ovulation and that their secretory capacity was ceased basically. The disappearance of a large number of granulosa cells was primarily caused by intracellular mitochondrial apoptosis and ER stress, while the regression of the theca layer resulted from the combination of effects from Beclin1-induced autophagy and caspase-induced apoptosis (Fig. 8). In addition, the different rates of regression between the granulosa and theca layer indicates that the granulosa cells of POFs maintains longer biological functions ahead of regression.

## MATERIALS AND METHODS

### Animals

Hyline white hens (*Gallus domesticus*) were raised in a local commercial farm and subjected to conventional breeding management conditions, with free access to feed and water, and under a controlled photoperiod of 14 hours light: 10 hours dark. All procedures were performed in accordance with the Guiding Principles for the Care and Use of Laboratory Animals of Zhejiang University.

### Tissue collection

Ovaries were collected 1 h after an expected ovulation and POFs (POF1-POF6) were isolated from chickens of approximately 300 days old which had laid six or more eggs in a sequence. The POFs were washed more than three times in PBS to remove red blood cells as make them as clean as possible. The POFs (POF1 to POF4) tissues were cut open and the granulosa layer was mechanically dissected using cell scrapers. Parts of the POFs were fixed for morphological observation and the other POFs or granulosa and theca layers from the POFs were used for WB or qRT-PCR analysis.

### Morphological observation

All tissues were fixed in formalin-acetic acid-alcohol (FAA) for 24 h at 4°C, subsequently embedded in paraffin, and sectioned to a thickness of 4 μm. HE staining was performed following standard histological procedures [38]. IF staining was performed as previously described [39]. The primary antibodies used for the IF were as follows: mouse anti-GRP78 (1:100, sc-376768, Santa Cruz Biotechnology, Santa Cruz, USA), rabbit anti-BCL2 (1:50, ab32124, Abcam, Cambridge, UK), mouse anti-Beclin1 (1:50, NBP1-00085) rabbit anti-p62 (1:200, NBP1-49955, Novus, Littleton, USA) and FOXL2 (1:50, 19672-1-AP, Proteintech, Wuhan, China). Sections were viewed under an Eclipse 80i microscope (Nikon, Tokyo, Japan). The fluorescence images of the slides were visualized using an IX70 fluorescence microscope (OLYMPUS, Tokyo, Japan). The tissue stained without adding primary antibody was considered to be a negative control.

### Oil Red O staining

Tissues were fixed in 4% paraformaldehyde and dehydrated in 15% and 30% sucrose, respectively. The fixed POFs were immersed in OCT embedding compound (6502, Thermo Scientific, Waltham, USA) and snap frozen. Frozen tissue was sectioned into 10 μm thickness using a cryostat (CryoStar NX50, Thermo Scientific, Waltham, USA). The sections were stained with a 0.3% Oil Red O solution (0.3 g in 100 ml isopropanol; Sigma-Aldrich, St. Louis, USA) for 15 min at room temperature. Nuclear staining was conducted using hematoxylin then photographed using an Eclipse 80i microscope.

### TUNEL assay

The ovarian fragment sections were incubated with the reagent in the terminal deoxynucleotidyl transferase-mediated deoxyuridine triphosphate nick-end labeling (TUNEL) BrightGreen Apoptosis Detection Kit (A112-03, Vazyme, Nanjing, China) according to the manufacturer’s instruction. Fluorescence images of the slides were visualized using an IX70 fluorescence microscope.

### Transmission electron microscopy

TEM was performed as previously described [40]. Briefly, the samples were fixed in 2.5% glutaraldehyde overnight at 4°C, post-fixed for 1.5 h in buffered 1% osmium tetroxide, dehydrated in a graded series of ethyl alcohol or acetone, and embedded in LX 112 epoxy resin. Ultrathin sections (70-90 nm) were cut on a Leica EM UC7 ultramicrotome (Leica Microsystems GmbH, Wetzlar, Germany) and mounted on formvar-coated copper grids. The samples were stained with 8% aqueous uranyl acetate and Reynold’s lead citrate, then observed and photographed on an Tecnai G2 Spirit (FEI Company, Hillsboro, USA) with an acceleration voltage of 120 kV at various magnifications.

### Western blot

Proteins were extracted using an ice-cold RIPA lysis buffer (P00138, Beyotime, Nanjing, China) with proteinase inhibitor (ST506, Beyotime, Nanjing, China). Equal amounts of proteins were measured using an BCA protein assay kit (A045-3, Jiancheng, Nanjing, China). Proteins were separated by electrophoresis and then electronically transferred to polyvinylidene fluoride membranes (Merck Millipore, Billerica, USA) using a BioRad system (BioRad, Hercules, USA). The membrane was blocked in 5% skimmed milk at room temperature for 2 h and subsequently incubated overnight at 4°C with corresponding primary antibodies, rabbit anti-BCL2 (1:200), mouse anti-GRP78 (1:200), rabbit anti-LC3β (1:100, sc-28266, Santa Cruz Biotechnology, Santa Cruz, USA), rabbit anti-CYP11A1 (1:200, CSB-PA006389LA01HU, CusAb, Wuhan, China), mouse anti-BAX (1:100, ab5714) and mouse anti-β-actin (1:1000, ab8226, Abcam, Cambridge, UK). Horseradish peroxidase-conjugated goat anti-rabbit or anti-mouse IgG (sc-2004 or sc-2005, Santa Cruz Biotechnology, Dallas, USA) were then used to detect proteins using Clarity ECL Western Blot Substrate kits (BioRad, Hercules, USA) and exposed using a ChemiScope 3400 Mini machine (Clinx, Shanghai, China). Protein quantification was performed using densitometry analyses on Quantity One Software.

### RNA extraction and qRT-PCR

Total RNA was extracted using a Trizol reagent (Invitrogen, Carlsbad, USA). RNA concentrations were measured using a NanoDrop 2000c (Thermo Scientific, Waltham, USA). The cDNA was generated from 2 μg total RNA using a HiScript II 1st Strand cDNA Synthesis Kit (R211-02, Vazyme, Nanjing, China) according to the manufacturer’s protocol. qRT-PCR was performed using HiScript II One Step qRT-PCR SYBR Green Kit (Q311-02, Vazyme, Nanjing, China). The 2^−ΔΔCt^ formula method was used to calculate relative fold-change values between samples. The primers used are provided in Table1.

**Table 1:** Sequences of the primers for qRT-PCR.

Gene name	Accession number	Primer sequence (5’-3’)	Product size (bp)	
*Bok*	AF290888.1	GAACATCTCGCTGCACTCG AAGGTCTTGCGGACAAACTC		207
*Bak1*	NM_001030920.1	ATGGACCCGGAGATCATGGA		103
		CGTACCGCTTGTTGATGTCG		
*Grp78*	NM_205491.1	GAATCGGCTAACACCAGAGGA		118
		CGCATAGCTCTCCAGCTCATT		
*Grp94*	NM_204289.1	CTGTTACTGCCAGCCACCA		186
		TCCACCTTTGCATCCAGGTCA		
*Atf4*	AB013138.1	TGAGCCTCTTGAACAACGAG		298
		TGTTCCATACCTAACAGGGC		
*Cyp11a1*	NM_001001756.1	CAAGACATGGCGTGACCA		131
		TGAAGAGGATGCCCGTGT		
*Hsd3b2*	XM_015294359.1	CAGCTGCTCTGGGAAGTCA		128
		GGGTCACCCCTGCAGTTT		
		ACGTGAAATACGCTGGAGGA		
β-actin	NM_205518	ACACCCACACCCCTGTGATGAA		136
		TGCTGCTGACACCTTCACCATTC		

### Measurement of P_4_ level

To measure the level of P_4_, the F1 and POFs (POF1-POF5) were homogenized in ice-cold 5% PBS and P_4_ levels were detected using BCA kit and ARCHITECT Progesterone Reagent Kit (Abbott, Lisnamuck, Ireland) according to the manufacturer’s protocol.

### Statistical analysis

All data were expressed as the means ± standard error of the means (SEM) and analyzed by One-way analysis of variance (ANOVA) with LSD and Duncan’s multiple-range tests using the SPSS16.0 software. *P* < 0.05 was considered as a statistically significant difference.
